# A revised landslide inventory of the Campania region (Italy)

**DOI:** 10.1038/s41597-023-02155-6

**Published:** 2023-06-05

**Authors:** Francesco Fusco, Rita Tufano, Pantaleone De Vita, Diego Di Martire, Mariano Di Napoli, Luigi Guerriero, Florindo Antonio Mileti, Fabio Terribile, Domenico Calcaterra

**Affiliations:** 1 Department of Civil and Environmental Engineering, Politecnico di Milano, Piazza Leonardo da Vinci 32, 20133 Milan, Italy; 2 CRISP Research Center, University of Naples Federico II, Via Università 100, 80055, Portici, Naples, Italy; 3 Department of Earth, Environment and Resources Sciences, University of Naples Federico II, Complesso Universitario di Monte Sant’Angelo, Via Cinthia 21, 80126 Naples, Italy; 4 SINTEMA Engineering srl, Spin Off University of Naples Federico II, Via Toledo 156, 80138 Naples, Italy; 5 WhereTech srl, Via Giacomo Peroni 400, 00131 Rome, Italy; 6 Department of Agriculture, University of Naples Federico II, Via Università 100, 80055, Portici, Naples, Italy

**Keywords:** Natural hazards, Databases

## Abstract

Landslides represent a severe geohazard in many countries. The availability of inventories depicting the spatial and temporal distribution of landslides is crucial for assessing landslide susceptibility and risk for territorial planning or investigating landscape evolution. Nevertheless, these inventories are usually affected by limitations due to their nonpublic availability and inhomogeneities in characterization and mapping. Such problems are fully recognizable by the analysis of the multiple landslide inventories of the Campania region, which is one of the Italian regions with the highest exposure to landslide hazard and risk. On this basis, a revised Landslide Inventory of the Campania region (LaICa), resulting from the processing of multiple existing landslide inventories, has been reconstructed. It aims to (i) provide a new geodatabase that is able to overcome issues derived from the coexistence of multiple inventories and (ii) provide a methodological paradigm able to support the reorganization of existing official inventories. The implication of LaICa, with its 83,284 records, will possibly improve the assessment of landslide susceptibility and then reassess the related risk.

## Abstract (summary)

**Table:** 

Measurement(s)	landslide
Technology Type(s)	Data Set

## Background & Summary

Landslides are complex natural phenomena representing severe geohazards in many countries^1^. For this reason, knowledge of the spatial and temporal distribution of landslides is crucial for the assessment of related hazards to support a comprehensive risk assessment. It is also of high importance for the analysis of landscape evolution and sediment budget from slope to basin scales.

Landslide inventories constitute a detailed register of both the spatial distribution and characteristics of past landslides^2,3^. Inventory maps can be created for different and multiple purposes^4^: landslide documentation from the slope or catchment scale^5–10^ to regional^11–17^ or national scales^18–24^; as a preliminary step for the assessment of landslide susceptibility, hazard and risk^25–34^; to investigate the landslide distribution, types and patterns related to morphological and geological factors^35–37^; and to study landscape evolution^38–43^. However, the real usefulness of these inventories is rather limited due to their common spatial inhomogeneity or use of different mapping methods and classification criteria^2^. Moreover, accessing a freely downloadable landslide geodatabase is usually the major restriction for studies aimed at landslide susceptibility and risk assessment. Such issues strongly affect the Campania region, which is one of the Italian regions with the highest percentage of area that is prone to landslide hazards (approximately 60%) and with the highest number of people exposed to landslide hazards, estimated to reach 302,783^44^. Despite this, a unique public and homogeneous landslide inventory covering the entire Campanian territory still does not exist, owing to the large number of public authorities that have been in charge of recognizing and mapping landslide and flood hazards and risks. Specifically, from 1998 to 2016, seven Basin Authorities (BAs) (http://www.difesa.suolo.regione.campania.it/content/category/6/26/38/), managing different zones of the Campania territory, carried out this activity with different criteria. After 2016, the BAs were incorporated as Units of Management (UoMs) in the Southern Apennine Hydrological District (SAHD; www.distrettoappenninomeridionale.it), a public authority in charge of regulating territorial planning under the safeguard against landslides and floods, and they collected previous landslide maps and made them publicly available in a repository of .pdf files on a web platform. In addition to these official landslide inventories, which currently represent the regulatory reference for territorial planning, the only common and homogeneous regional landslide geodatabase is the national project called IDROGEO (https://idrogeo.isprambiente.it/app/iffi/r/17), which reports landslides surveyed within the IFFI project (Italian Landslide Inventory^20^), accounting for approximately 23,500 records in the Campania region.

In such a framework, the implementation of a consistent and homogeneous landslide geodatabase is intended to i) provide a new geodatabase able to overcome issues deriving from the coexistence of multiple inventories and ii) provide a methodological paradigm able to support the reorganization of existing official inventories at a national scale. The implication of the Landslide Inventory of the Campania region (LaICa) will possibly improve the assessment of landslide susceptibility, hazard zoning and the related risk.

To this end, a revised LaICa resulting from the homogenization of existing records has been reconstructed and is presented here. A similar approach to implement a unique regional landslide inventory was adopted by the Piemonte (SIFRAP - https://webgis.arpa.piemonte.it/Geoviewer2D/?config=other-configs/SIFRAP_config.json) and Emilia Romagna regions (https://ambiente.regione.emilia-romagna.it/it/geologia/geologia/dissesto-idrogeologico/la-carta-inventario-delle-frane).

Developing the LaICa geodatabase has been conceived, promoting an improved assessment of landslide susceptibility and hazard zoning at different scales, from municipal to regional, in the Campania region, as well as supporting research activities and interdisciplinary collaborations.

The LaICa geodatabase was funded by the H2020 LandSupport (LS) (www.landsupport.eu) project, which aims to develop a geoSpatial DSS (S-DSS) open-access platform to be used for guiding land policies within EU countries under the 2030 UN Sustainable Development Goals, including those related to climate change.

## Methods

**Fig. 1:**
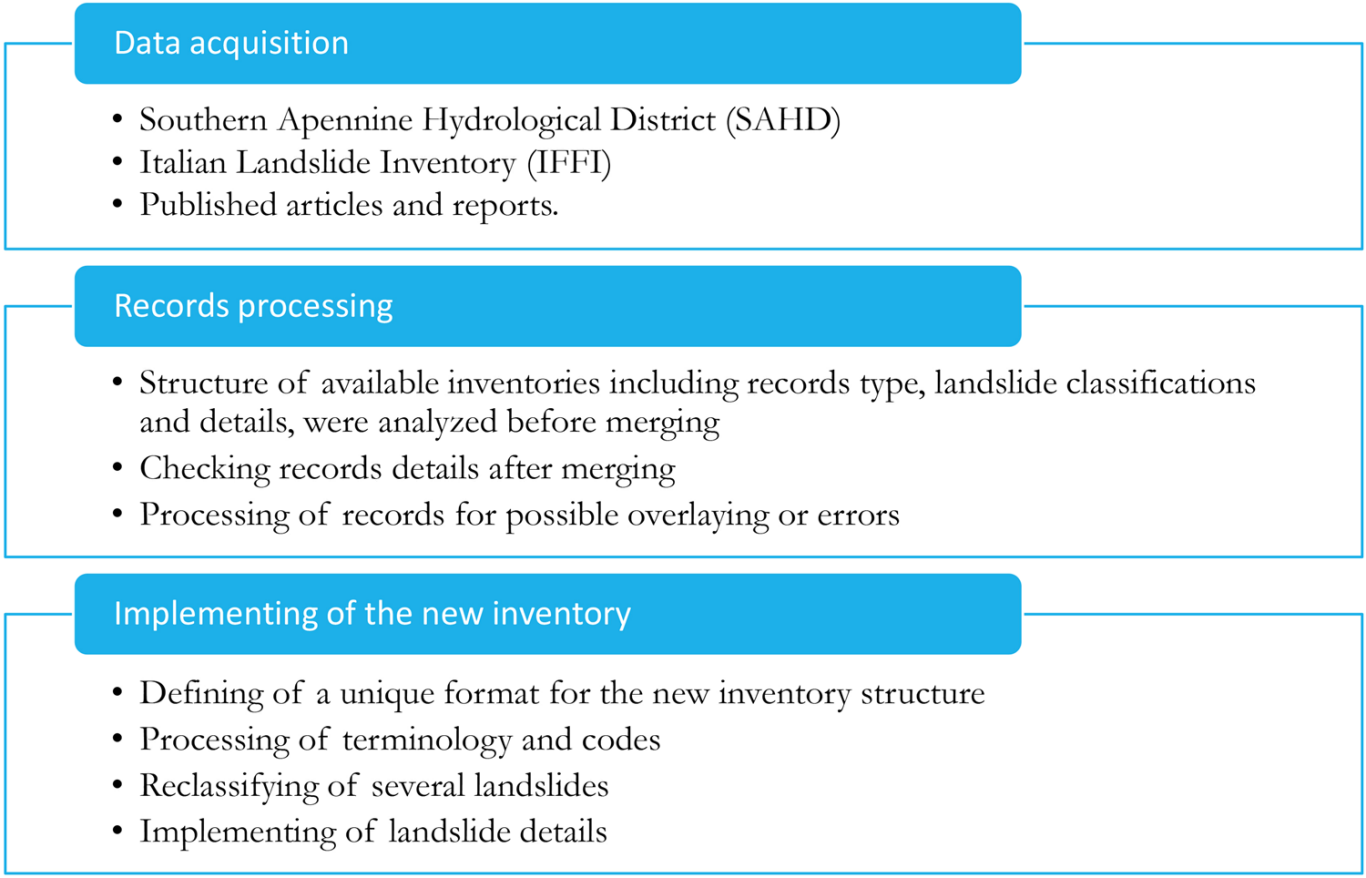
Workflow of the approach used to implement the LaICa inventory.

The development of the landslide inventory followed three steps: (i) analysis of available databases; (ii) processing of geometries and attributes; and (iii) implementation of the new landslide database, including technical validation and quality assessment. A workflow of these steps is illustrated in Fig. 1.

### Data acquisition

Landslide geodatabases for the Campania region were collected from three sources of data: (1) public domain data gathered from the seven Unit of Managements (UoMs) of SAHD, corresponding to the former BAs; (2) public domain data from the Italian Landslide Inventory (IFFI); and (3) literature data, including scientific articles and reports regarding some inventories of high-magnitude landslide events. The structures of available inventories, such as the methods used for data extraction, collection and filing, were different and are described below.

### Public domain inventories

**Fig. 2:**
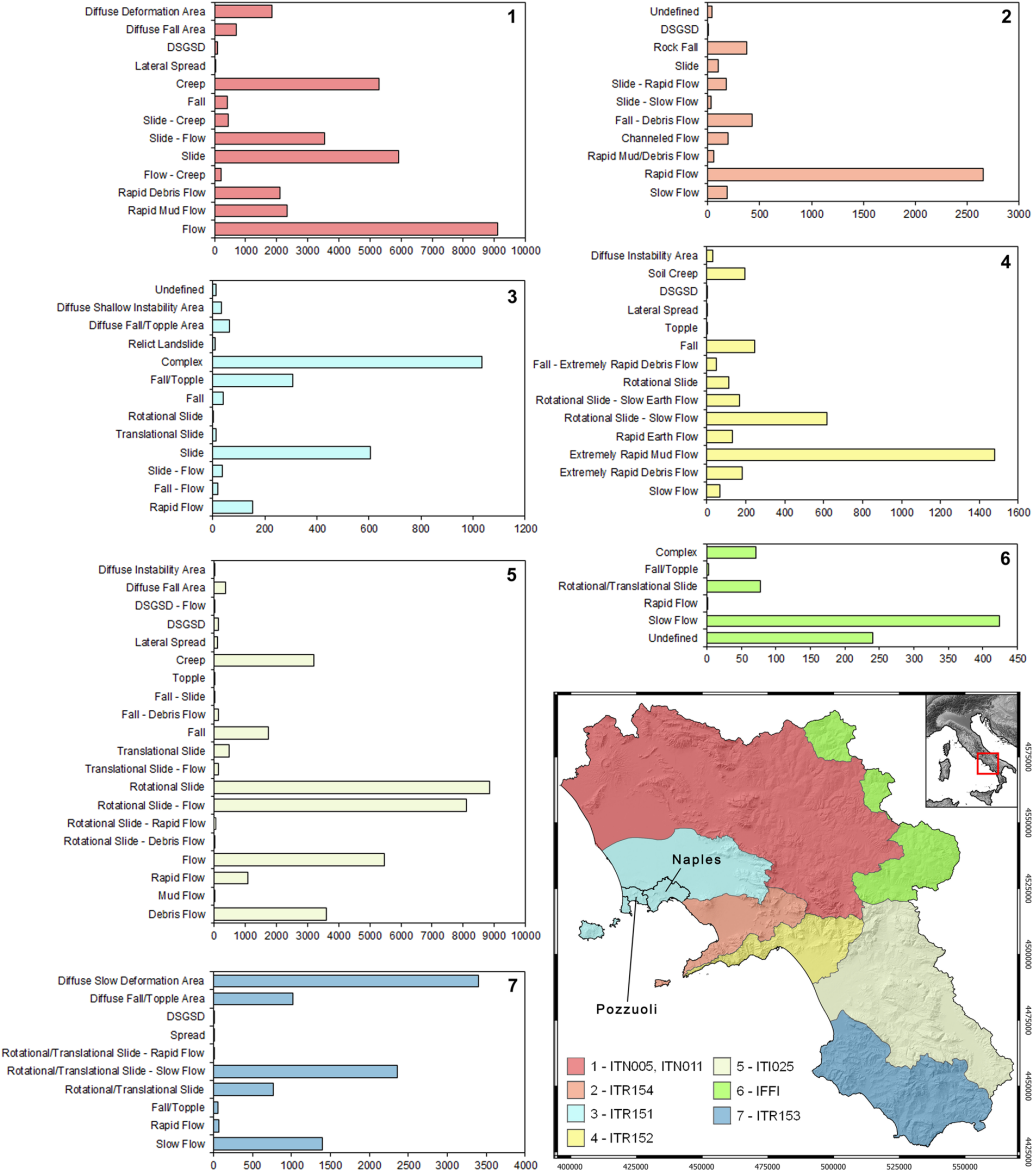
Extension of the seven UoMs covering the Campania territory. Comparison of landslide types 1 to 7 recognized in the landslide geodatabase reconstructed by UoMs and considered for implementing the LaICa inventory. *Key words*: UoM Volturno (ITN011); UoM Liri-Garigliano (ITN005); UoM Campania Nord Occidentale (ITR151); UoM Sarno (ITR154); UoM Destra Sele (ITR152); UoM Sinistra Sele (ITR153); and UoM Interregionale Sele (ITI025); Italian Landslides Inventory (IFFI). Records from published articles and reports related to Naples and Pozzuoli inventories, were implemented into the UoM ITR151 database.

A deep element-by-element analysis and homogenization processing were carried out before merging all databases in the LaICa inventory. Such analyses were achieved with great effort due to the considerable number of mapped geometries (records) as well as to different inventory structures, as described below. In detail, records collected for the SAHD landslide inventories by the seven former UoMs cover 92% of the Campania region (Fig. 2): UoM Volturno (ITN011); UoM Liri-Garigliano (ITN005); UoM Campania Nord Occidentale (ITR151); UoM Sarno (ITR154); UoM Destra Sele (ITR152); UoM Sinistra Sele (ITR153); and UoM Interregionale Sele (ITI025). It is worth noting that records obtained from ITN005 and ITN011 are dated to 2011 and mapped at a scale of 1:25,000, while all the other records are dated to 2016, with a scale of 1:5,000. Unfortunately, no records from SAHD inventories are available for the remaining areas of the Campania territory (8%). Concerning the IFFI inventory (http://www.difesa.suolo.regione.campania.it/; Fig. 2), records cover the entire regional territory and were mapped until 2006 at a scale of 1:25,000. This inventory was realized through a collaboration between Campania Region public authorities and the National Geological Service (now the Italian National Institute for Environmental Protection and Research, ISPRA). Actually, it represents the official reference for the Campania region, as well as for the entire national territory. Both IFFI and UoM geodatabases comprise features such as polygons or dots associated with the records and an alphanumerical code containing details of the records. However, linear features also characterize several records of the IFFI inventory. Polygonal features depict landslides with areal extents equal to or greater than 1 ha, while dots depict landslides of lower extensions or landslides with poor information. All the considered geodatabases were recognized as having different structures for landslide feature types and details and considering differentiated classification criteria.

### Published scientific articles and reports

Further data known from published scientific papers, technical reports and published scientific articles were also considered, although limited to very small areas of the Campania region within the UoM ITR151 administrative boundary (Fig. 2). The inventory was implemented according to the 2013 directive of the Campania Region public authority (Regional Council, n. 146 of 27/05/2013). According to this directive, each municipality of the region was prepared and invited to carry out detailed studies regarding the different natural hazards affecting the territories in the framework of the Municipal Emergency Plans (MEPs) of Civil Protection, such as the case of Palma Campania (Naples Province) municipality^45^. In detail, approximately 70 landslides that were mapped in the Municipality of Pozzuoli (Naples Province) from 2013 to February 2020 were considered in LalCa. Finally, landslide inventories implemented for the city of Naples, derived from published articles^46–48^, were considered. In this case, 1,322 landslides that occurred from 1816 to 2020 were mapped and implemented in a geodatabase following the same structure as the ITR151. All new data were merged and implemented in the ITR151 geodatabase.

### Quality of existing landslide inventories

The heterogeneity of the structure of landslide geodatabases considered in this study (Fig. 2), as well as the approach used to characterize their records, required the assessment of the information quality by means of three criteria (Table 1): (1) landslide classification; (2) type and quantity of details considered for the landslide event; and (3) possibility to specify the detachment/source, transit, and accumulation landslide areas. The quality of records, which are affected by the classification that was used, was estimated by considering whether a single or mixed classification was adopted. This analysis revealed that approximately 81.6% of records (69,698) were characterized by the use of mixed landslide classification to define the movement type, while the remaining 18.4% of records (15,673) were characterized by single landslide classification. Subsequently, a degree of quality (low, medium, or high) was attributed to each inventory, considering the quantity of information available for each record, such as the landslide velocity, state, style, distribution, and damage. Only for 14.5% of records (12,368) was the high quality of details attributed to the availability of more than three pieces of information, including the velocity, state, style, and distribution or damage. A medium degree of quality was assigned to 42% of records (35,890), and a low degree of quality was assigned to the remaining 43.5% (37,113) due to the presence of two or one details, including “state of activity” and “velocity” or “distribution” in the first case and “state of activity” or “velocity” in the second case. Finally, the possibility of specifying the source and transit/accumulation areas of a landslide was evaluated based on the specific degree of quality (low, medium, or high). From such an analysis, databases are mainly characterized by low quality because no landslide zoning data were available. Only two landslide geodatabases, representing 18% of the total records, reached a high quality level. The element-by-element comparison between the SAHD and IFFI inventories revealed inconsistencies in terms of the total number of inventoried landslides and their perimetration. Due to the mapping accuracy of records and their type of representation (dots, polygons), UoM records are more accurate than IFFI. Thus, the SAHD landslide inventories were considered primarily to implement the LaICa due to their official and regulatory role in any territorial planning activity. Therefore, to obtain a complete inventory for the entire territory of Campania (≈1,359,903 ha), only where SAHD inventories are not available (≈112,090 ha, ≈8.2%), the IFFI was assumed to be the single official representative inventory (as shown in Fig. 2). Consequently, the risk of an incomplete number and representation of records was inevitably accepted (only ≈1% of total records). Accordingly, landslide inventories published in scientific papers and mostly related to high-magnitude and catastrophic landslide events were considered to increase the database significance (0.8% of total records). Unfortunately, the same approach cannot be considered for the remaining IFFI records that do not overlap with the SAHD records. Such a decision is based on the uncertainty of several missed or incorrectly/differently detected and positioned IFFI records (see the Technical Validation paragraph for more details).

**Table 1:** Quality of records collected from UoM Volturno (ITN011); UoM Liri-Garigliano (ITN005); UoM Campania Nord Occidentale (ITR151); UoM Sarno (ITR154); UoM Destra Sele (ITR152); UoM Sinistra Sele (ITR153); and UoM Interregionale Sele (ITI025); Italian Landslide Inventory (IFFI); Publ. data (published articles and reports).

Inventory ID	Classification		Details			Zoning		
	Single	Mixed	Low	Medium	High	Low	Medium	High
ITN05-11		×	×					×
ITR151	×			×		×		
ITR154	×		×				×	
ITR152		×			×	×		
ITR153		×		×				×
ITI025	×				×	×		
IFFI		×	×			×		
Publ. data	×			×			×	

*Classification*: type of landslide classification used if single (one type) or mixed (more than one type); *Details*: information about landslides (among them, the state, style, distribution, etc.); *Zoning*: possibility to specify source and transit/accumulation areas of a landslide.

## Data Records

**Fig. 3:**
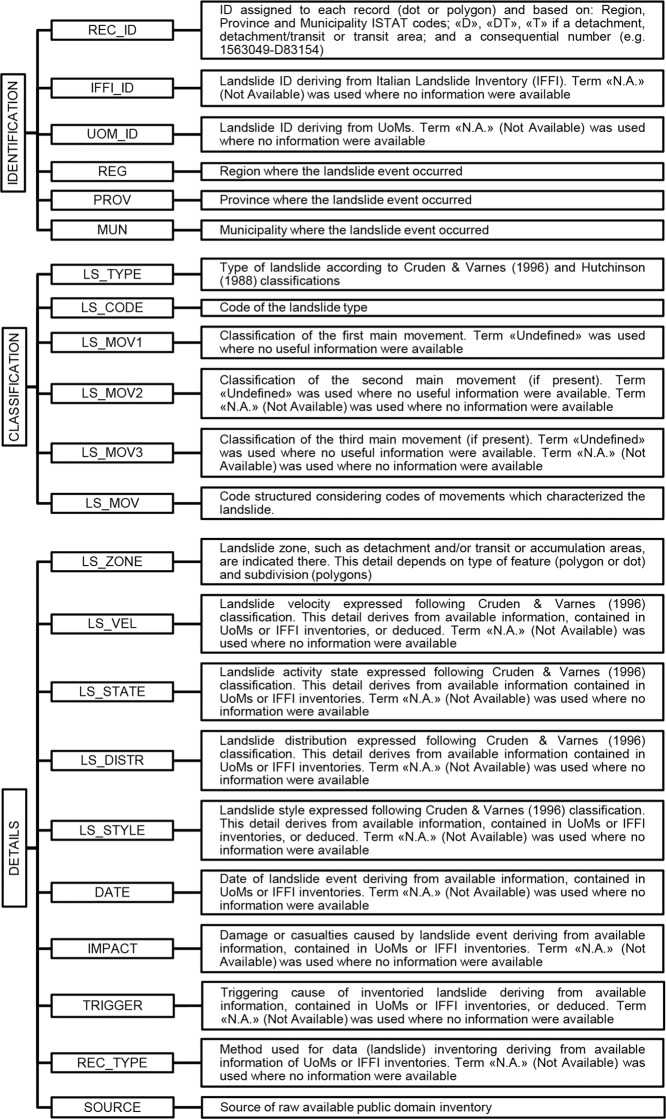
Structure of the LaICa alphanumerical database with all fields described.

The LaICa inventory includes 83,284 total landslide records distributed over the Campania region and is characterized by a combined cartographic and alphanumeric geodatabase. Available for download at Figshare^49^ in comma-separated values (CSV) file format or shapefile, the inventory is proposed as a unique reference for landslide analyses in a geographical information system (GIS) environment. In detail, the LaICa geodatabase was implemented by an accurate check of features (polygons or dots) associated with records, which was accomplished after a merging procedure aimed at checking possible overlays or errors. As previously described, due to the different structures of the existing alphanumerical geodatabases, the main phase of this work was the definition of a unique, coherent and homogeneous format. To this end, a 22-field database was implemented concerning three fundamental groups of recorded information (Fig. 3): landslide identification, classification, and details. In detail, the structure of the alphanumerical database is described below:

(A) *Landslide identification information*, including six fields (Fig. 3): “REC_ID”, “IFFI_ID”, “UOM_ID”, “REG”, “PROV”, and “MUN”. A new ID was assigned to records (geometry) by merging ISTAT (Italian national STATistical institute) codes of the region, province and municipality, landslide zone and an incremental number. For example, a record with the ID “1565138-DT01132” means: ‘15’ = Region’s code for Campania; ‘65’ = Province’s code; ‘138’ = Municipality’ code; DT = landslide zone; and 01132 = polygons’ incremental number. To this aim, a preliminary lack of geographical information existing for ≈80% of records was fixed through the intersection of the LaICa cartographical database with the administrative boundaries (available from sit2.regione.campania.it/content/dati-di-base) in a GIS environment. Finally, specific fields related to the landslide ID in the original database (UoM or IFFI), available only for ≈55% of records, were also implemented in the new geodatabase to create links to official databases still in force.
(B) *Landslide classification information*, including six fields (Fig. 3): “LS_TYPE”, “LS_CODE”, “LS_MOV1”, “LS_MOV2”, “LS_MOV3”, and “LS_MOV”. According to the Hutchinson (1988)^50^ and Cruden & Varnes (1996)^51^ classifications, the type of landslide and related movement (one or more), as well as codes, were revised, and initial records were rearranged considering eleven main groups (Table 2). Records coinciding with “fall”, “topple”, “slide”, “creep” and “deep-seated gravitational slope deformation” (Fig. 2) were grouped more easily due to no significant terminological heterogeneity (Table 2). In fact, in these cases, terms were associated only with landslide and movement types. Otherwise, records concerned with the “flow” landslide type (≈44% of total) showed greater heterogeneity (more than 30 types) (Fig. 2 and Table 2). In this case, information regarding the type of material involved, landslide velocity and possible multiple evolutionary stages were moved to other fields, grouping records based on terms related to landslide type. In cases where two or more types of movements were recognized as characterizing a record (for example, a slide evolving into a slow flow, a fall evolving into a debris flow, etc.), the main movement was considered as classifying the entire landslide record (for example, a fall evolving into a debris flow: event characterized by a fall as the first movement, and evolving into debris flow as the second movement) (Table 2). Furthermore, records comprising “complex” landslide types (≈1.3% of total) were processed, and both the type and movement were reinterpreted (Fig. 2). However, where information concerning the type of landslide or movement is lacking, the term “undefined” (UNDF) was used. Finally, records coinciding with areas characterized by diffuse shallow landslides or diffuse deformation (≈9% of total) were implemented as “Diffuse Shallow Instability Area” and “Diffuse Deformation Area”, respectively (Table 2). In all fields, when information was not available, the term N.A. (i.e., Not Available) was used.

**Table 2:** Criterion of classification of landslide movements and types, according to the Hutchinson (1988)^50^ and Cruden & Varnes (1996)^51^ classifications. Grouped landslide types from UoMs and IFFI inventories are also shown. *Key words*: Rotat., Rotational; Trans., Translational; Gravit., Gravitational; Def., Deformation; Diff., Diffuse; Inst., Instability.

Landslide group		Movement		Reclassified and grouped landslide movement
Type	Code	Type	Code	
FALL	FLL	Fall	FLL	Fall; Rock Fall
TOPPLE	TPL	Topple	TPL	Topple
FALL/TOPPLE	FLL/TPL	Fall/Topple	FLL/TPL	Fall/Topple
SLIDE	SLD	Rotat. Slide	RSLD	Slide; Rotat. Slide; Trans. Slide; Rotat. Slide/Trans. Slide; Trans./Rotat. Slide
		Trans. Slide	TSLD	
		Rotat. Slide/Trans. Slide	RSLD/TSLD	
FLOW	FLW	Debris Flow	DFLW	Slow Flow; Rapid Flow; Rapid Mud Flow; Rapid Mud/Debris Flow; Rapid Debris Flow; Rapid Earth Flow; Extremely Rapid Debris Flow; Extremely Rapid Mud Flow; Debris Flow; Mud Flow; Flow; Channeled Flow; Slide - Flow; Slide - Slow Flow; Slide - Rapid Flow; Rotat. Slide - Debris Flow; Rotat. Slide - Rapid Flow; Rotat. Slide - Flow; Rotat. Slide - Slow Flow; Rotat. Slide - Slow Earth Flow; Trans. Slide - Flow; Rotat./Trans. Slide - Slow Flow; Rotat./Trans. Slide - Rapid Flow; Flow - Creep; Fall - Extremely Rapid Debris Flow; Fall - Debris Flow; Fall - Flow; Fall - Extremely Rapid Debris Flow
		Earth Flow	EFLW	
		Flow	FLW	
SPREAD	SPD	Spread	SPD	Spread; Lateral Spread
CREEP	CRP	Soil Creep	SCRP	Soil Creep; Mass Creep; Deep Creep; Creep
		Mass Creep	MCRP	
DEEP SEATED GRAVIT. SLOPE DEF.	DSGSD	Undefined	DSGSD	Deep Seated Gravitational Slope Deformation; Deep Seated Gravitational Slope Deformation - Flow
UNDEFINED	UNDF	Undefined	UNDF	Undefined; Relict Landslides
DIFF. SHALLOW INST. AREA	A-SHW	Fall	FLL	Diffuse Fall/Topple Area; Diffuse Shallow Instability Area; Diffuse Instability Area; Diffuse Fall Area
		Topple	TPL	
		Slide	RSLD/TSLD	
		Flow	FLW	
DIFF. DEFORMATION AREA	A-DEF	Undefined	UNDF	Diffuse Slow Deformation Area; Diffuse Deformation Area
(C) *Landslide details*, including 10 fields (Fig. 3): “LS_ZONE”, “LS_VEL”, “LS_STATE”, “LS_DISTR”, “LS_STYLE”, “DATE”, “IMPACT”, “TRIGGER”, “REC_TYPE”, and “SOURCE”. As previously described, landside zoning, such as detachment and transit or accumulation areas, is not usually specified in the original records. For this reason, a careful analysis was carried out in a GIS environment by adding a specific field (Fig. 3). The terms “detachment” or “transit” were assigned to those records coinciding with one or more features (polygon or dot) indicating where the landslide started (source area) or transited/stopped, respectively. Otherwise, for records associated with a single feature where no possibility to identify source or transit/accumulation areas exists, the term “detachment/transit” was introduced. However, more details about this field are discussed in the “Technical validation” paragraph. Information on the fields “landslide velocity”, “state of activity”, “style” and “distribution”, as previously described was not available for all records and was mainly associated with the landslide type field (Table 2). Thus, some of these details, such as “velocity” and “style”, were assessed in several cases by expert judgment and assigned to specific fields (Fig. 3), according to the Cruden & Varnes (1996)^51^ classification. Available information associated with a landslide event regarding damage, human injuries or casualties and triggering cause (i.e., rainfall, earthquakes, etc.) was also included. Furthermore, the types of approaches used for landslide inventorying (e.g., aerial photointerpretation, field survey or archive) were also indicated. Finally, the original source of information was filed into a specific field (Fig. 3). In all cases with no available information, the term N.A. (i.e., Not Available) was used.

## Technical Validation

In comparison with the preceding landslide geodatabases, the LaICa can be appraised for the improvement in the (1) accuracy of records; (2) homogenization of existing official landslide geodatabases; and (3) reliability of the inventory in the framework of an improved reanalysis of landslide susceptibility from municipal to regional scales.

### Homogeneity and completeness of records

The new landslide inventory of the Campania territory is created by the merging and homogenization of previously existing public records, which were reconstructed by the seven former UoMs and IFFI project by the adoption of different criteria. Such inhomogeneity of landslide geodatabases is recognized as the major limitation in the usage of information, especially for zones located across boundaries of different UoMs. A relevant problem with preexisting landslide geodatabases is also the frequent use of the Italian language. Therefore, although this aspect could be considered secondary, the LaICa inventory was implemented in English, thus in accordance with the original classification terms. Specifically, the main improvement of the new geodatabase was the homogenization of details in the records, according to the Hutchinson (1988^50^) and Cruden & Varnes (1996^51^) classifications. This was also intended as useful for promoting a wider diffusion of the new landslide geodatabase in the national and international scientific community. As a result, ≈95% of records were associated with a specific landslide type (Table 2), while only the remaining ≈5% were unclassifiable (undefined) due to the unavailability of information. Furthermore, for 14% of records coinciding with complex landslides, the first type of movement was not specified due to a lack of information. Concerning other landslide details (Fig. 3), such as “landslide velocity”, “style”, “distribution”, and “state of activity”, the rearrangement of available information allowed fully detailed records to be obtained for 15% of records, with only 3% characterized by a total lack of information. Moreover, the information that is most lacking is the date of the landslide event and details about damage and/or casualties and triggering causes. Indeed, this information was available or inferred for only less than 3% of all records. Due to its structure, the LaICa geodatabase could be considered a reference for implementing landslide geodatabases in other regions affected by similar inconsistencies in existing landslide inventories, which were collected, similar to the Campania region, by other hydrographic districts. In such a view, the structure and approach used by the LaICa would be replicated, favoring analyses of a unique and homogeneous landslide inventory.

### Accuracy evaluation through landslide events

**Fig. 4:**
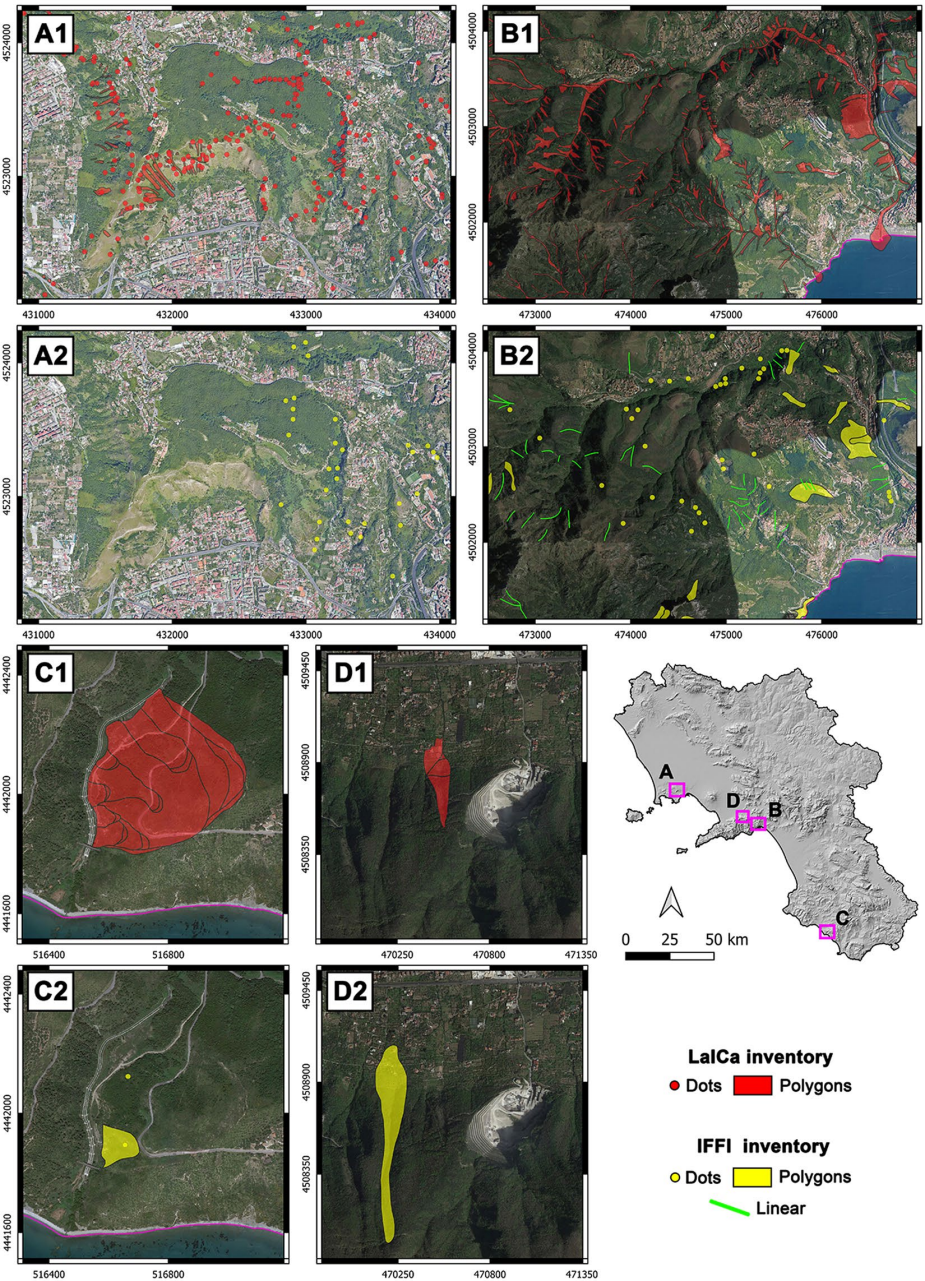
Comparison between LaICa and IFFI inventories to evaluate the accuracy of records through cases of well-documented landslide events described in scientific publications and/or governmental reports, with regard to: the i) number of events; ii) geometric representation (as dot, polygon or line); and iii) positioning and shape of geometries. A, the case of Camaldoli Hill (Municipality of Naples) landslide events^44,46–48^; B, the case of Lattari Mts. (within the boundary of the Province of Salerno) high-magnitude landslide events^9,10^; C, the case of Rizzico landslide events (Province of Salerno)^52^; D, the case of the Nocera inferiore event (Province of Salerno)^53^.

To evaluate the accuracy of representative LaICa areas in the Campania region, well-documented landslide events described in scientific publications and/or governmental reports were considered for comparison with those reported in the IFFI geodatabase. In Fig. 4, we present four case studies showing that LaICa significantly increases the accuracy of records in terms of i) the number of records (landslides); ii) the geometric representation of records (dot, polygon or line); and iii) the positioning and shape of records. The first case of landslide events, which occurred in the Camaldoli Hill area^44,46–48^ (Municipality of Naples), shows a major LaICa accuracy and completeness in terms of inventoried records (Fig. 4 - A1, A2). In fact, in this area, 434 total records are inventoried by the LaICa, while the IFFI geodatabase reports a total of only 32. This significant change is due to the occurrence of many landslide events after 2006, which are considered by the LaICa geodatabase. The second case is the landslide events that occurred in October 1954 in a sector of the Lattari Mts.^9,10^, including Cava de’ Tirreni, Vietri and Cetara municipalities (Province of Salerno), and this case shows the greater accuracy of LaICa records (Fig. 4 - B1, B2) in comparison to the IFFI geodatabase (230 records instead of 97, respectively). Moreover, landslides inventoried by the IFFI are mainly represented by dots or lines instead of polygons. This aspect indicates the major accuracy of the LaICa geodatabase in estimating areas affected by landslide events, whose total equals 184 ha in the LaICa and only 25.5 ha in the IFFI. Finally, the cases of the Rizzico landslide^52^ (Fig. 4 - C1, C2) and the Nocera Inferiore landslide^53^ (Fig. 4 - D1, D2), both of which occurred in Salerno Province, demonstrate the accuracy of LaICa records in terms of the positioning and shape of geometries. The filing of these representative and very well-known case studies reveals how the LaICa has increased the number of landslide events not recognized by the IFFI geodatabase.

### Overall coverage and representativeness of records

**Fig. 5:**
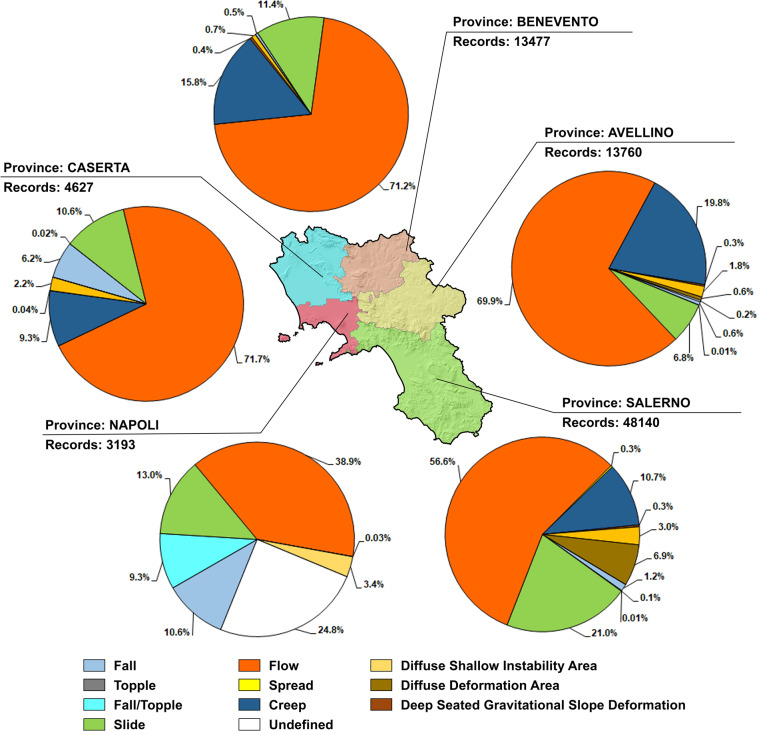
Number of records and percentage of landslide types for the five provinces of the Campania region.

**Fig. 6:**
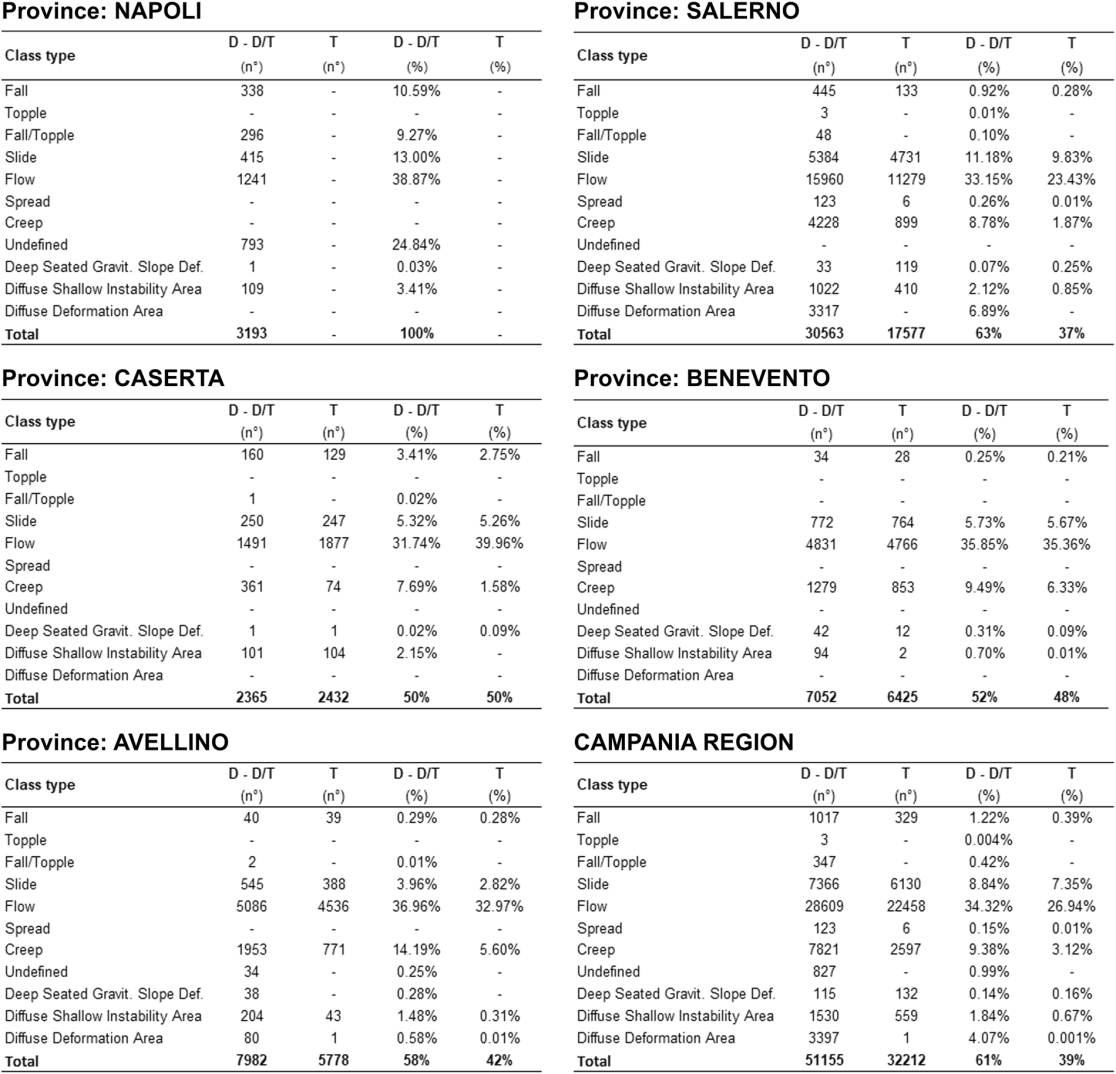
Number and percentages of landslide events divided by the type and areas in the Campania region and its provinces. *Key words*: D) detachment; D/T) detachment/transit; T) transit; Gravit.) gravitational; Def.) deformation.

In the framework of landslide susceptibility and/or risk assessment, the availability of a high-quality landslide geodatabase is undoubtedly a fundamental achievement. For the Campania region, the homogenization and integration of records of eight preexisting landslide inventories into the LaICa geodatabase allowed a drastic reduction in the related limitations. Furthermore, due to its structure, the LaICa geodatabase provides the possibility for users to perform research and execute different types of analysis, from regional to municipal scales, in an easier and more consistent way; examples include the analysis of specific types of landslides or their distribution in selected areas, such as administrative boundaries or geologically homogeneous areas. In this regard, a first test analysis was carried out for the Campania region and its five provinces (Napoli, Caserta, Salerno, Avellino and Benevento) that obtained percentages and numbers of records coinciding with specific landslide classes (Table 2; Fig. 5). A second test was performed by counting landslide events, from the province to regional scales, and their areal extension, based on detachment and detachment/transit areas (Fig. 6). As a result, 51,155 landslides were recognized for the whole Campania region (61% of total records), with 3.4 events/km^2^ (1.7 from IFFI) extending over ≈141,911 ha (96,806 ha from IFFI) and corresponding to 10.4% of the regional territory; these events can be grouped as follows: ≈1% detachment, ≈42% detachment/transit, and 57% transit/accumulation. In addition to the underestimation of unstable areas due to the association of records with dots or small polygons, the accuracy of landslide susceptibility and/or risk analysis carried out through the LaICa is also affected by the number of landslide events inventoried by the SAHD and IFFI geodatabases, which do not comprise recent landslide events. In this regard, we carried out a further analysis at the municipality scale to evaluate the representativeness of the LaICa compared to the IFFI inventory. In the Table S1 we summarize the estimated values of unstable areas for each affected municipality of the Campania region (457 of 550 total). The analysis revealed how values for 296 municipalities (65% of total) are affected by underestimation when the IFFI was considered, while only for 160 (35% of total) when considering LaICa.

### LaICa limitations

Based on the approach used to implement the LaICa, some technical limitations need to be stated. The LaICa inventory resulted from processing different preexisting landslide inventories, comprising records gathered on different dates: 2006 for IFFI, 2016 for UoMs and 2020 for information from scientific articles and reports. Thus, the LaICa was not homogeneously updated over the Campania region. Such a condition is also due to the limitation of merging ≈1,500 IFFI records that do not overlap with SAHD records. In fact, further subjective, nonreplicable analyses are required to achieve this goal. In this regard, it must be mentioned that LaICa records were processed based on landslide classification and detailing, adopting a unique and uniform criterion. No geometries (dots or polygons) were modified in terms of extension, shape and/or positioning to preserve the integrity of raw records and replicability of our analyses. Thus, the presence of some cases of errors in the geometric representation was inevitably accepted. Furthermore, the inventories from UoMs, scientific articles and reports were initially implemented for the reconstruction of landslide susceptibility maps. Based on the adopted approach, in the case of two events inventoried in the same location, the latest event prevails over the first event so that the same landslide area is not counted twice. This aspect mostly coincides with large phenomena, such as deep-seated gravitational slope deformations (DSGSDs) or diffuse deformation areas. Finally, some technical limitations caused by a lack of information affecting some fields of LaICa records may exist, especially for specific analyses focused on landslide hazards or risk assessments.

## Usage Notes

### Data repository structure

The LaICa geodatabase is available in the repository^49^. It is intended to be open and available to all landslide researchers and professional users. Data generated in this work are accessible from the repository in which a single packaged zip archive named “LaICa_database” was uploaded. It contains a “READ_ME” text file that provides guidance information for users and two subfolders: “LaICa_Metafiles” and “LaICa_Inventory”. The contents of the files and subfolders within the zip archive are described below:

(A) LaICa_Metafiles contains two CSV files, “LaICa_Metafile_Polygons” and “LaICa_Metafile_Points”, which provide a summary of all LaICa records attributes (see the Data records section).
(B) LaICa_Inventory contains the cartographical/alphanumerical geodatabase divided into two SHAPEFILES: “LaICa_polygons” and “LaICa_points”. The first shapefile contains 81,216 features (polygons), while the second contains 2,068 (points). All features are projected in the WGS 84/UTM zone 33 N coordinate system.

The majority of the work was carried out by QGIS v.3.16.4, but all items were produced in a format importable into any other GIS software. Despite the high number of features mapped, there are many more landslide occurrences that can be recognized. Further versions of the LaICa database will integrate other information derived from field surveys, including new landslide phenomena.

## Supplementary information

Supplementary Table S1

## Data Availability

No customized code was produced to prepare or analyze the dataset because software that works in a GIS environment is required to open and process the LaICa dataset. Specifically, we used and suggest the open-source QGIS software available in different releases at https://qgis.org/it/site/. However, further specific software (open-source or licensed) is required to open and process CSV files associated with the LaICa database.
