# Supplementary material for: A revised landslide inventory of the Campania region (Italy)
Source: Sci Data. 2023 Jun 5;10:355. doi: 10.1038/s41597-023-02155-6 (PMC10241882; doi:10.1038/s41597-023-02155-6)
Supplement: Supplementary file 1 — Supplementary Table S1 [file 41597_2023_2155_MOESM1_ESM.pdf]

Estimation of unstable areas				
	Total		Underestimation	
**Municipality**	**LaICa**	**IFFI**	**LaICa**	**IFFI**
Acerno	64.2	6.5		90%
Agerola	61.4	18.4		70%
Agropoli	509.0	315.1		38%
Aiello del Sabato	90.6	100.2	10%	
Ailano	53.2	32.0		40%
Airola	24.4	27.7	12%	
Albanella	517.5	0.0		100%
Alfano	247.5	158.3		36%
Alife	54.5	48.7		11%
Altavilla Irpina	179.1	162.0		10%
Altavilla Silentina	538.2	1.4		100%
Alvignano	189.8	177.6		6%
Amalfi	33.1	46.0	28%	
Anacapri	0.0	70.3	100%	
Andretta	188.4	387.4	51%	
Angri	26.7	0.0		100%
Apice	600.3	541.6		10%
Apollosa	558.2	550.6		1%
Aquara	870.0	308.0		65%
Aquilonia	1111.1	1160.7	4%	
Ariano Irpino	2285.0	2294.9	0%	
Arienzo	1.3	1.0		20%
Arpaia	10.0	37.0	73%	
Arpaise	220.4	189.5		14%
Ascea	610.1	260.3		57%
Atena Lucana	20.5	0.0		100%
Atrani	1.6	0.0		100%
Atripalda	62.3	73.5	15%	
Auletta	1274.5	208.4		84%
Avella	15.4	7.8		49%
Avellino	104.5	115.3	9%	
Bacoli	0.1	3.1	98%	
Bagnoli Irpino	136.7	105.4		23%
Baia e Latina	41.1	50.5	19%	
Baiano	15.3	0.0		100%
Barano d'Ischia	24.7	95.1	74%	
Baronissi	32.6	29.5		9%
Baselice	996.3	847.4		15%
Battipaglia	3.3	0.0		100%
Bellona	1.3	0.0		100%
Bellosguardo	558.1	41.7		93%
Benevento	864.4	783.7		9%
Bisaccia	436.8	539.7	19%	
Bonea	152.2	89.8		41%
Bonito	286.0	165.6		42%
Boscotrecase	0.0	11.7	100%	
Bracigliano	82.6	55.5		33%

Bucciano	52.0	36.2		30%
Buccino	2824.4	0.0		100%
Buonabitacolo	17.7	12.5		29%
Buonalbergo	665.7	737.4	10%	
Caggiano	1713.8	199.2		88%
Caianello	29.4	8.7		70%
Caiazzo	484.4	429.9		11%
Cairano	86.8	97.5	11%	
Calabritto	948.5	175.3		82%
Calitri	320.2	491.3	35%	
Calvanico	237.7	0.0		100%
Calvi	59.4	34.3		42%
Calvi Risorta	52.3	19.3		63%
Camerota	504.6	135.9		73%
Camigliano	19.8	27.3	27%	
Campagna	1731.5	71.4		96%
Campolattaro	426.5	395.7		7%
Campoli del Monte Taburno	404.3	383.5		5%
Campora	426.0	1.9		100%
Candida	150.9	147.4		2%
Cannalonga	429.4	358.1		17%
Capaccio	444.2	343.5		23%
Caposele	2206.3	759.1		66%
Capri	0.0	35.8	100%	
Capriati a Volturno	128.5	106.6		17%
Capriglia Irpina	194.1	90.0		54%
Capua	32.2	36.5	12%	
Carbonara di Nola	3.4	0.0		100%
Carife	131.2	75.4		43%
Carinola	23.7	14.2		40%
Casagiove	0.3	0.0		100%
Casalbore	1101.5	1104.4	0%	
Casalbuono	4.3	0.0		100%
Casalduni	849.0	833.5		2%
Casaletto Spartano	1067.7	268.9		75%
Casalvelino	350.8	310.3		12%
Casamarciano	0.1	0.0		100%
Casamicciola Terme	75.0	203.1	63%	
Caselle in Pittari	1304.2	678.8		48%
Caserta	169.3	190.2	11%	
Casola di Napoli	2.3	8.9	74%	
Cassano Irpino	154.9	83.4		46%
Castel Baronia	113.3	96.3		15%
Castel Campagnano	186.7	176.1		6%
Castel di Sasso	162.0	173.3	7%	
Castel Morrone	75.1	103.7	28%	
Castel S. Giorgio	18.1	0.0		100%
Castel San Lorenzo	358.4	97.4		73%
Castelcivita	1065.8	9.6		99%
Castelfranci	376.5	269.3		28%

Castelfranco in Miscano	1153.7	1263.8	9%	
Castellabate	427.6	300.5		30%
Castellammare di Stabia	11.3	28.8	61%	
Castello Matese	138.1	56.0		59%
Castelnuovo Cilento	303.6	227.5		25%
Castelnuovo di Conza	780.8	86.4		89%
Castelpagano	798.7	703.5		12%
Castelpoto	185.1	197.9	6%	
Castelvenere	6.5	6.3		3%
Castelvetere In Val Fortore	255.9	357.0	28%	
Castelvetere sul Calore	434.2	349.5		20%
Castiglione del Genovesi	30.4	35.5	14%	
Cautano	169.7	148.7		12%
Cava de' Tirreni	234.4	23.7		90%
Celle di Bulgheria	1321.0	635.7		52%
Centola	1526.3	1010.9		34%
Ceppaloni	581.7	667.9	13%	
Ceraso	818.5	680.0		17%
Cerreto Sannita	901.2	836.0		7%
Cervinara	361.2	295.2		18%
Cervino	2.2	2.9	27%	
Cesinali	6.7	27.9	76%	
Cetara	38.1	18.7		51%
Chianche	80.9	70.1		13%
Chiusano San Domenico	355.1	301.4		15%
Cicerale	353.0	403.9	13%	
Ciorlano	55.7	35.4		36%
Circello	1283.9	1319.7	3%	
Colle Sannita	666.8	586.8		12%
Colliano	1371.5	73.8		95%
Conca dei Marini	2.6	4.9	46%	
Conca della Campania	46.2	32.3		30%
Contrada	31.9	48.7	35%	
Controne	81.5	0.0		100%
Contursi Terme	618.5	5.7		99%
Conza della Campania	184.6	196.3	6%	
Corbara	132.6	0.0		100%
Corleto Monforte	353.0	256.0		27%
Cuccaro Vetere	352.0	90.7		74%
Cusano Mutri	1289.8	938.1		27%
Domicella	3.3	0.5		86%
Dragoni	13.8	9.5		31%
Dugenta	7.7	2.8		64%
Durazzano	20.3	14.9		27%
Eboli	177.0	0.0		100%
Ercolano	22.0	257.8	91%	
Faicchio	102.5	41.3		60%
Falciano del Massico	7.2	3.6		50%
Felitto	911.1	152.3		83%
Fisciano	116.7	4.5		96%

Flumeri	330.8	263.7		20%
Foglianise	81.8	57.2		30%
Foiano di Val Fortore	674.5	774.2	13%	
Fontanarosa	62.4	40.2		36%
Fontegreca	83.4	80.6		3%
Forchia	2.5	9.4	73%	
Forino	29.0	15.9		45%
Forio	492.9	538.8	9%	
Formicola	123.7	117.8		5%
Fragneto l'Abate	548.2	619.4	11%	
Fragneto Monforte	570.8	598.3	5%	
Francolise	8.1	5.8		29%
Frasso Telesino	344.0	311.3		10%
Frigento	890.7	600.6		33%
Furore	3.8	12.0	69%	
Futani	406.4	229.2		44%
Gallo Matese	134.3	68.8		49%
Galluccio	88.2	81.5		8%
Gesualdo	373.7	209.1		44%
Giano Vetusto	80.8	101.6	20%	
Giffoni Sei Casali	108.8	60.3		45%
Giffoni Valle Piana	445.5	274.8		38%
Ginestra degli Schiavoni	690.6	673.6		2%
Gioi	822.3	604.0		27%
Gioia Sannitica	396.3	311.4		21%
Giungano	222.6	143.4		36%
Gragnano	77.2	33.6		57%
Greci	220.2	377.7	42%	
Grottaminarda	150.5	101.5		33%
Grottolella	81.3	157.8	49%	
Guardia Lombardi	1040.9	857.8		18%
Guardia Sanframondi	342.3	325.3		5%
Ischia	3.8	28.1	86%	
Ispani	151.6	97.3		36%
Lacco Ameno	0.0	92.0	100%	
Lacedonia	832.4	775.5		7%
Lapio	279.5	279.3		0%
Laureana Cilento	460.3	399.7		13%
Laurino	1775.1	313.0		82%
Laurito	529.4	154.6		71%
Lauro	22.5	56.7	60%	
Laviano	890.8	35.9		96%
Letino	316.9	187.2		41%
Lettere	29.3	0.0		100%
Liberi	18.5	49.7	63%	
Limatola	145.1	134.5		7%
Lioni	342.8	200.5		41%
Luogosano	136.3	127.8		6%
Lustra	391.8	275.8		30%
Maddaloni	3.4	9.1	63%	

Magliano Vetere	448.1	277.6		38%
Maiori	212.0	77.5		63%
Manocalzati	142.4	124.3		13%
Marano di Napoli	0.6	0.0		100%
Marzano Appio	53.4	13.3		75%
Marzano di Nola	2.4	15.8	85%	
Massa di Somma	0.0	0.0		
Massa Lubrense	102.7	128.0	20%	
Melito Irpino	311.1	385.7	19%	
Melizzano	215.5	224.0	4%	
Mercato S. Severino	148.9	0.0		100%
Mercogliano	99.5	78.4		21%
Meta di Sorrento	0.0	17.8	100%	
Mignano Monte Lungo	155.0	156.2	1%	
Minori	13.4	6.4		52%
Mirabella Eclano	147.3	115.7		21%
Moiano	56.7	27.0		52%
Moio della Civitella	609.4	653.1	7%	
Molinara	761.2	575.9		24%
Mondragone	38.4	36.6		5%
Montaguto	173.3	154.2		11%
Montano Antilia	948.2	509.3		46%
Monte di Procida	0.1	14.4	99%	
Monte San Giacomo	681.5	159.8		77%
Montecalvo Irpino	1312.7	1613.9	19%	
Montecorice	188.8	251.9	25%	
Montecorvino Pugliano	493.8	44.1		91%
Montecorvino Rovella	314.3	110.3		65%
Montefalcione	187.2	265.1	29%	
Montefalcone di Val Fortore	1272.9	1185.9		7%
Monteforte Cilento	656.6	455.1		31%
Monteforte Irpino	53.7	74.8	28%	
Montefredane	110.4	106.4		4%
Montefusco	148.1	134.6		9%
Montella	260.4	271.7	4%	
Montemarano	492.3	469.9		5%
Montemiletto	263.3	261.2		1%
Montesano sulla Marcellana	97.7	23.0		76%
Montesarchio	349.1	459.6	24%	
Monteverde	614.3	647.6	5%	
Montoro	132.0	6.8		95%
Morcone	1791.3	1854.2	3%	
Morigerati	608.9	328.4		46%
Morra De Sanctis	574.0	601.5	5%	
Moschiano	27.4	55.5	51%	
Mugnano del Cardinale	12.8	1.8		86%
Napoli	26.5	5.9		78%
Nocera Inferiore	99.2	11.3		89%
Nocera Superiore	87.3	0.0		100%
Novi Velia	551.3	456.5		17%

Nusco	304.5	292.4		4%
Ogliastro Cilento	276.3	293.0	6%	
Olevano sul Tusciano	84.9	47.0		45%
Oliveto Citra	1513.8	126.1		92%
Omignano	119.5	104.0		13%
Orria	374.5	374.7	0%	
Ospedaletto D'Alpinolo	74.3	65.9		11%
Ottati	376.4	7.5		98%
Ottaviano	49.3	172.9	71%	
Padula	118.5	60.6		49%
Paduli	1095.0	808.4		26%
Pagani	16.9	0.0		100%
Pago del Vallo di Lauro	3.3	0.0		100%
Pago Veiano	1056.9	887.7		16%
Palma Campania	12.7	3.1		75%
Palomonte	474.8	0.0		100%
Pannarano	195.5	191.0		2%
Paolisi	52.3	44.5		15%
Parolise	54.1	68.8	21%	
Paternopoli	285.4	217.3		24%
Paupisi	181.5	130.0		28%
Pellezzano	22.6	17.2		24%
Perdifumo	878.9	656.2		25%
Perito	344.8	339.6		1%
Pertosa	88.9	6.3		93%
Pesco Sannita	508.7	582.9	13%	
Petina	466.7	0.0		100%
Petruro Irpino	64.4	108.9	41%	
Piaggine	262.4	145.2		45%
Piana di Monte Verna	143.0	144.5	1%	
Piano di Sorrento	10.8	99.0	89%	
Piedimonte Matese	81.3	51.2		37%
Pietradefusi	70.1	78.8	11%	
Pietramelara	74.7	65.3		13%
Pietraroja	762.2	762.1		0%
Pietrastornina	366.7	283.4		23%
Pietravairano	35.2	35.1		0%
Pietrelcina	260.4	336.8	23%	
Pignataro Maggiore	5.7	3.6		37%
Pimonte	9.0	9.3	3%	
Pisciotta	533.5	272.1		49%
Pollena Trocchia	0.0	7.7	100%	
Polla	20.8	0.0		100%
Pollica	464.7	569.9	18%	
Ponte	244.5	233.9		4%
Pontecagnano Faiano	1.9	0.0		100%
Pontelandolfo	429.9	485.9	12%	
Pontelatone	97.3	119.5	19%	
Positano	32.1	70.8	55%	
Postiglione	1950.9	415.4		79%

Pozzuoli	4.5	2.2		52%
Praiano	10.1	33.4	70%	
Prata di Principato Ultra	40.5	50.9	21%	
Prata Sannita	94.9	73.3		23%
Pratella	94.7	76.4		19%
Pratola Serra	38.2	92.3	59%	
Presenzano	59.7	48.5		19%
Prignano Cilento	255.8	209.8		18%
Procida	2.8	7.5	62%	
Quadrelle	5.2	1.8		66%
Quarto	0.1	0.0		100%
Quindici	120.3	114.7		5%
Ravello	36.5	21.4		41%
Raviscanina	82.0	51.4		37%
Reino	801.3	823.9	3%	
Riardo	7.1	6.1		14%
Ricigliano	175.8	0.0		100%
Rocca d'Evandro	378.5	204.8		46%
Rocca San Felice	383.0	347.2		9%
Roccabascerana	259.0	274.9	6%	
Roccadaspide	1104.5	5.7		99%
Roccagloriosa	1633.7	477.9		71%
Roccamonfina	35.1	0.0		100%
Roccapiemonte	19.4	0.0		100%
Roccarainola	8.5	19.9	57%	
Roccaromana	149.8	150.0	0%	
Rocchetta e Croce	117.9	152.1	22%	
Rofrano	1429.3	864.2		40%
Romagnano al Monte	289.9	0.0		100%
Roscigno	727.9	479.5		34%
Rotondi	29.5	25.4		14%
Rutino	271.2	211.3		22%
Ruviano	253.8	241.7		5%
S. Egidio del Monte Albino	64.9	0.0		100%
Sacco	671.2	414.6		38%
Sala Consilina	38.2	0.0		100%
Salento	386.1	272.9		29%
Salerno	234.0	70.8		70%
Salvitelle	291.9	0.0		100%
Salza Irpina	4.6	20.7	78%	
San Bartolomeo In Galdo	898.1	1016.2	12%	
San Cipriano Picentino	185.4	131.1		29%
San Felice a Cancello	12.7	11.7		8%
San Giorgio del Sannio	65.1	80.1	19%	
San Giorgio la Molara	1662.5	2605.9	36%	
San Giovanni a Piro	1123.9	575.6		49%
San Giuseppe Vesuviano	33.6	0.3		99%
San Gregorio Magno	493.9	11.5		98%
San Gregorio Matese	493.7	187.2		62%
San Leucio del Sannio	86.6	123.1	30%	

San Lorenzello	67.6	25.4		62%
San Lorenzo Maggiore	415.5	295.6		29%
San Lupo	358.8	429.8	17%	
San Mango Piemonte	35.5	41.4	14%	
San Mango sul Calore	366.2	413.9	12%	
San Marco dei Cavoti	861.7	1278.8	33%	
San Martino Sannita	73.3	59.3		19%
San Martino Valle Caudina	192.3	139.7		27%
San Mauro Cilento	186.3	312.2	40%	
San Mauro la Bruca	424.5	303.3		29%
San Michele di Serino	25.4	40.0	36%	
San Nazzaro	24.8	23.9		4%
San Nicola Baronia	132.4	112.6		15%
San Nicola Manfredi	90.6	101.4	11%	
San Pietro al Tanagro	34.9	0.0		100%
San Pietro Infine	104.5	111.2	6%	
San Potito Sannitico	18.5	9.1		51%
San Potito Ultra	32.7	40.8	20%	
San Prisco	58.9	39.4		33%
San Rufo	117.0	0.0		100%
San Sossio Baronia	384.3	335.5		13%
Santa Croce del Sannio	191.6	308.9	38%	
Santa Lucia di Serino	14.6	10.6		27%
Santa Maria a Vico	0.1	0.0		100%
Santa Marina	312.8	340.7	8%	
Santa Paolina	74.7	91.6	18%	
Sant'Agata dei Goti	508.3	561.1	9%	
Sant'Agnello	16.4	46.6	65%	
Sant'Anastasia	0.0	25.3	100%	
Sant'Andrea di Conza	21.2	43.3	51%	
Sant'Angelo a Cupolo	65.1	65.3	0%	
Sant'Angelo a Fasanella	217.5	106.0		51%
Sant'Angelo a Scala	123.8	129.5	4%	
Sant'Angelo all'Esca	17.8	24.5	27%	
Sant'Angelo d'Alife	21.0	19.2		9%
Sant'Angelo dei Lombardi	515.1	472.5		8%
Sant'Arcangelo Trimonte	96.5	169.6	43%	
Sant'Arsenio	1.5	0.0		100%
Santo Stefano del Sole	68.2	73.5	7%	
Santomenna	283.2	10.9		96%
Sanza	817.5	330.1		60%
Sapri	248.6	77.9		69%
Sarno	273.2	268.8		2%
Sassano	135.7	2.1		98%
Sassinoro	247.9	235.0		5%
Savignano Irpino	145.3	286.5	49%	
Scala	62.9	46.5		26%
Scampitella	100.8	45.7		55%
Senerchia	1274.8	325.5		74%
Serino	280.4	370.2	24%	

Serramezzana	75.0	106.7	30%	
Serrara Fontana	2.4	65.6	96%	
Serre	560.0	20.3		96%
Sessa Aurunca	363.1	289.3		20%
Sessa Cilento	265.7	376.9	30%	
Siano	33.0	38.3	14%	
Sicignano degli Alburni	1264.7	90.8		93%
Sirignano	1.2	0.3		74%
Solofra	127.6	8.8		93%
Solopaca	185.5	205.7	10%	
Somma Vesuviana	0.0	21.9	100%	
Sorbo Serpico	23.5	32.3	27%	
Sorrento	19.5	60.5	68%	
Sparanise	0.0	0.1	100%	
Sperone	1.6	0.0		100%
Stella Cilento	279.4	297.3	6%	
Stio	450.3	345.0		23%
Sturno	100.4	163.8	39%	
Summonte	93.6	115.1	19%	
Taurano	1.7	10.5	84%	
Taurasi	200.5	212.5	6%	
Teano	35.1	20.8		41%
Teggiano	679.5	0.0		100%
Teora	134.0	139.1	4%	
Terzigno	0.0	104.6	100%	
Tocco Caudio	417.2	557.6	25%	
Tora e Piccilli	17.0	16.8		1%
Torchiara	199.5	279.2	29%	
Torella dei Lombardi	293.4	274.9		6%
Torraca	395.9	122.0		69%
Torre del Greco	45.7	373.0	88%	
Torre le Nocelle	40.1	58.5	32%	
Torre Orsaia	651.8	532.2		18%
Torrecuso	493.6	620.8	20%	
Torrioni	17.1	26.9	37%	
Tortorella	377.2	215.2		43%
Tramonti	220.9	42.8		81%
Trecase	0.0	2.7	100%	
Trentinara	354.1	421.8	16%	
Trevico	307.9	177.5		42%
Tufino	0.2	0.0		100%
Tufo	41.7	67.8	38%	
Vairano Patenora	181.8	182.1	0%	
Vallata	519.3	457.0		12%
Valle Agricola	132.6	99.0		25%
Valle dell'Angelo	121.2	0.0		100%
Valle di Maddaloni	11.9	21.6	45%	
Vallesaccarda	277.7	230.2		17%
Vallo della Lucania	543.3	498.1		8%
Valva	962.4	0.2		100%

Venticano	44.9	68.1	34%	
Vibonati	396.4	162.0		59%
Vico Equense	26.9	109.1	75%	
Vietri sul Mare	138.2	30.4		78%
Villamaina	148.9	159.8	7%	
Villanova del Battista	380.6	431.5	12%	
Visciano	1.2	2.7	56%	
Vitulano	312.2	398.5	22%	
Volturara Irpina	84.5	112.0	25%	
Zungoli	322.6	201.1		38%
**Total (n)**	**141911**	**96748**	**160**	**296**
**Total (%)**	**10.4%**	**7.1%**	**35%**	**65%**

Estimation of unstable areas				
	Total		Underestimation	
**Municipality**	**LaICa**	**IFFI**	**LaICa**	**IFFI**
65001	64.2	6.5		90%
63003	61.4	18.4		70%
65002	509.0	315.1		38%
64001	90.6	100.2	10%	
61001	53.2	32.0		40%
62001	24.4	27.7	12%	
65003	517.5	0.0		100%
65004	247.5	158.3		36%
61002	54.5	48.7		11%
64002	179.1	162.0		10%
65005	538.2	1.4		100%
61003	189.8	177.6		6%
65006	33.1	46.0	28%	
63004	0.0	70.3	100%	
64003	188.4	387.4	51%	
65007	26.7	0.0		100%
62003	600.3	541.6		10%
62004	558.2	550.6		1%
65008	870.0	308.0		65%
64004	1111.1	1160.7	4%	
64005	2285.0	2294.9	0%	
61004	1.3	1.0		20%
62005	10.0	37.0	73%	
62006	220.4	189.5		14%
65009	610.1	260.3		57%
65010	20.5	0.0		100%
65011	1.6	0.0		100%
64006	62.3	73.5	15%	
65012	1274.5	208.4		84%
64007	15.4	7.8		49%
64008	104.5	115.3	9%	
63006	0.1	3.1	98%	
64009	136.7	105.4		23%
61006	41.1	50.5	19%	
64010	15.3	0.0		100%
63007	24.7	95.1	74%	
65013	32.6	29.5		9%
62007	996.3	847.4		15%
65014	3.3	0.0		100%
61007	1.3	0.0		100%
65015	558.1	41.7		93%
62008	864.4	783.7		9%
64011	436.8	539.7	19%	
62009	152.2	89.8		41%
64012	286.0	165.6		42%
63009	0.0	11.7	100%	
65016	82.6	55.5		33%

62010	52.0	36.2		30%
65017	2824.4	0.0		100%
65018	17.7	12.5		29%
62011	665.7	737.4	10%	
65019	1713.8	199.2		88%
61008	29.4	8.7		70%
61009	484.4	429.9		11%
64013	86.8	97.5	11%	
64014	948.5	175.3		82%
64015	320.2	491.3	35%	
65020	237.7	0.0		100%
62012	59.4	34.3		42%
61010	52.3	19.3		63%
65021	504.6	135.9		73%
61011	19.8	27.3	27%	
65022	1731.5	71.4		96%
62013	426.5	395.7		7%
62014	404.3	383.5		5%
65023	426.0	1.9		100%
64016	150.9	147.4		2%
65024	429.4	358.1		17%
65025	444.2	343.5		23%
64017	2206.3	759.1		66%
63014	0.0	35.8	100%	
61014	128.5	106.6		17%
64018	194.1	90.0		54%
61015	32.2	36.5	12%	
63015	3.4	0.0		100%
64019	131.2	75.4		43%
61017	23.7	14.2		40%
61018	0.3	0.0		100%
64020	1101.5	1104.4	0%	
65026	4.3	0.0		100%
62015	849.0	833.5		2%
65027	1067.7	268.9		75%
65028	350.8	310.3		12%
63018	0.1	0.0		100%
63019	75.0	203.1	63%	
65029	1304.2	678.8		48%
61022	169.3	190.2	11%	
63022	2.3	8.9	74%	
64021	154.9	83.4		46%
64022	113.3	96.3		15%
61023	186.7	176.1		6%
61024	162.0	173.3	7%	
61026	75.1	103.7	28%	
65034	18.1	0.0		100%
65035	358.4	97.4		73%
65030	1065.8	9.6		99%
64023	376.5	269.3		28%

62016	1153.7	1263.8	9%	
65031	427.6	300.5		30%
63024	11.3	28.8	61%	
61025	138.1	56.0		59%
65032	303.6	227.5		25%
65033	780.8	86.4		89%
62017	798.7	703.5		12%
62018	185.1	197.9	6%	
62019	6.5	6.3		3%
62020	255.9	357.0	28%	
64024	434.2	349.5		20%
65036	30.4	35.5	14%	
62021	169.7	148.7		12%
65037	234.4	23.7		90%
65038	1321.0	635.7		52%
61102	1526.3	1010.9		34%
62022	581.7	667.9	13%	
65040	818.5	680.0		17%
62023	901.2	836.0		7%
64025	361.2	295.2		18%
61028	2.2	2.9	27%	
64026	6.7	27.9	76%	
65041	38.1	18.7		51%
64027	80.9	70.1		13%
64028	355.1	301.4		15%
65042	353.0	403.9	13%	
61030	55.7	35.4		36%
62024	1283.9	1319.7	3%	
62025	666.8	586.8		12%
65043	1371.5	73.8		95%
65044	2.6	4.9	46%	
61031	46.2	32.3		30%
64029	31.9	48.7	35%	
65045	81.5	0.0		100%
65046	618.5	5.7		99%
64030	184.6	196.3	6%	
65047	132.6	0.0		100%
65048	353.0	256.0		27%
65049	352.0	90.7		74%
62026	1289.8	938.1		27%
64031	3.3	0.5		86%
61033	13.8	9.5		31%
62027	7.7	2.8		64%
62028	20.3	14.9		27%
65050	177.0	0.0		100%
63064	22.0	257.8	91%	
62029	102.5	41.3		60%
61101	7.2	3.6		50%
65051	911.1	152.3		83%
65052	116.7	4.5		96%

64032	330.8	263.7		20%
62030	81.8	57.2		30%
62031	674.5	774.2	13%	
64033	62.4	40.2		36%
61034	83.4	80.6		3%
62032	2.5	9.4	73%	
64034	29.0	15.9		45%
63031	492.9	538.8	9%	
61035	123.7	117.8		5%
62033	548.2	619.4	11%	
62034	570.8	598.3	5%	
61036	8.1	5.8		29%
62035	344.0	311.3		10%
64035	890.7	600.6		33%
65053	3.8	12.0	69%	
65054	406.4	229.2		44%
61038	134.3	68.8		49%
61039	88.2	81.5		8%
64036	373.7	209.1		44%
61040	80.8	101.6	20%	
65055	108.8	60.3		45%
65056	445.5	274.8		38%
62036	690.6	673.6		2%
65057	822.3	604.0		27%
61041	396.3	311.4		21%
65058	222.6	143.4		36%
63035	77.2	33.6		57%
64037	220.2	377.7	42%	
64038	150.5	101.5		33%
64039	81.3	157.8	49%	
64040	1040.9	857.8		18%
62037	342.3	325.3		5%
63037	3.8	28.1	86%	
65059	151.6	97.3		36%
63038	0.0	92.0	100%	
64041	832.4	775.5		7%
64042	279.5	279.3		0%
65060	460.3	399.7		13%
65061	1775.1	313.0		82%
65062	529.4	154.6		71%
64043	22.5	56.7	60%	
65063	890.8	35.9		96%
61044	316.9	187.2		41%
63039	29.3	0.0		100%
61045	18.5	49.7	63%	
62038	145.1	134.5		7%
64044	342.8	200.5		41%
64045	136.3	127.8		6%
65064	391.8	275.8		30%
61048	3.4	9.1	63%	

65065	448.1	277.6		38%
65066	212.0	77.5		63%
64046	142.4	124.3		13%
63041	0.6	0.0		100%
61050	53.4	13.3		75%
64047	2.4	15.8	85%	
63092	0.0	0.0		
63044	102.7	128.0	20%	
64048	311.1	385.7	19%	
62039	215.5	224.0	4%	
65067	148.9	0.0		100%
64049	99.5	78.4		21%
63046	0.0	17.8	100%	
61051	155.0	156.2	1%	
65068	13.4	6.4		52%
64050	147.3	115.7		21%
62040	56.7	27.0		52%
65069	609.4	653.1	7%	
62041	761.2	575.9		24%
61052	38.4	36.6		5%
64051	173.3	154.2		11%
65070	948.2	509.3		46%
63047	0.1	14.4	99%	
65075	681.5	159.8		77%
64052	1312.7	1613.9	19%	
65071	188.8	251.9	25%	
65072	493.8	44.1		91%
65073	314.3	110.3		65%
64053	187.2	265.1	29%	
62042	1272.9	1185.9		7%
65074	656.6	455.1		31%
64054	53.7	74.8	28%	
